# Laparoscopic resection of a pancreatic serous cystadenoma preserving the integrity of main pancreatic duct: a case report

**DOI:** 10.4076/1757-1626-2-8318

**Published:** 2009-07-16

**Authors:** Michail Pitiakoudis, Petros Zezos, Anastasia Oikonomou, Christos Tsalikidis, Georgios Kouklakis, Sotirios Botaitis, Constantinos Simopoulos

**Affiliations:** 1 2^nd^ Department of Surgery, Democritus University of Thrace, University General Hospital Dragana68100 AlexandroupolisGreece; 2 Gastrointestinal Endoscopy Unit, Democritus University of Thrace, University General Hospital Dragana68100 AlexandroupolisGreece; 3 Radiology Department, Democritus University of Thrace, University General Hospital Dragana68100 AlexandroupolisGreece

## Abstract

Pancreatic serous cystadenomas are rare benign cystic neoplasms. Extended operations are unnecessary for serous cystadenomas and minimally invasive surgery should be performed. Laparoscopic pancreatic procedures are under evaluation. We present a case of a 79-year-old Greek woman with symptomatic cholelithiasis and a serous pancreatic cystadenoma located at the neck of the pancreas. In the occasion of a standard laparoscopic cholecystectomy the pancreatic mass was resected with a novel minimally invasive laparoscopic method preserving the integrity of the main pancreatic duct and the whole pancreas. Laparoscopic resection is a feasible, safe and effective treatment of benign pancreatic tumors, in experienced hands under proper indications.

## Introduction

In the recent years, the advances in abdominal imaging techniques have resulted into an increasing frequency of pancreatic cystic neoplasms identification, which are considered to be rare. Pancreatic serous cystadenomas belong to pancreatic cystic neoplasms and are considered to have benign biological and clinical features and course. Serous cystadenomas account for about 25% of all cystic tumors of the pancreas [1,2].

Only relatively recently, Compagno and Oertel, described in 1978 the morphological and clinical features of this new entity, but there are still debating issues regarding the optimal management and treatment of these tumors [3].

Because it is difficult to establish a correct diagnosis of the benign nature of the lesion pre-operatively, some experts recommend resection of all pancreatic cystic neoplasms [4,5], whereas others propose a more selective approach recommending observation of the asymptomatic serous cystadenomas and resection of the large, symptomatic or indistinguishable from mucinous neoplams cases [6,7].

Still though, in clinical practice, in many cases serous cystadenomas are resected even when they were small and asymptomatic. Extended operations are unnecessary for serous cystadenomas, and minimally invasive surgery should be performed, because they are benign neoplasms with only a slight malignant potential [8].

Laparoscopic pancreatic procedures, including the role of laparoscopic surgery in patients with cystic neoplasms of the pancreas, are under evaluation. Laparoscopic spleen-preserving distal pancreatectomy for benign cystic neoplasms in the body or the tail of the pancreas is a safe and feasible option, in centers having experience in both pancreatic surgery and laparoscopic procedures [9].

## Case presentation

A 79-year-old Greek woman was referred to our department for the management of discomfort in the upper abdomen over the last 2-3 weeks, aggravated after food ingestion. The patient was not a smoker or an alcohol consumer and appeared healthy and well nourished. The physical examination and laboratory examinations including tumor markers were unremarkable.

**Figure 1.**
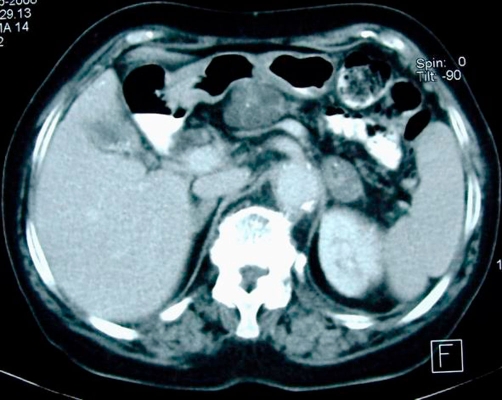
Contrast enhanced axial CT shows a well defined, lobulated cystic mass with internal septations at the neck of pancreas. Concomitant findings are cholelithiasis and an adenoma of the left adrenal.

**Figure 2.**
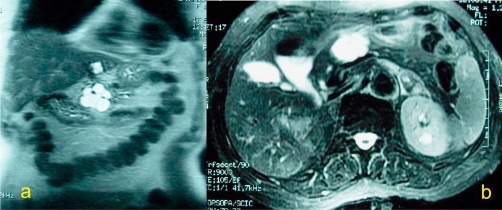
Coronal T2 weighted **(a)** and axial T2 weighted fat saturated **(b)** MRI reveal a multiloculated cystic mass at the pancreatic neck. A simple cyst at the left lobe of the liver is also seen at the coronal plane **(a)**.

**Figure 3.**
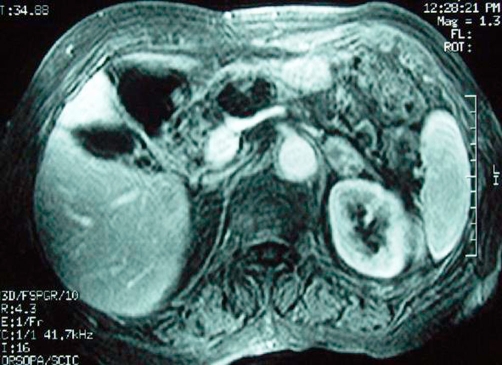
Gadolinium enhanced, fat saturated, gradient echo axial MRI disclose complete absence of enhancement of the mass at the pancreatic neck consistent with its cystic nature.

An abdominal ultrasound (US) showed multiple stones in the gallbladder without features of cholecystitis and a multilocular cystic mass in the neck of the pancreas, with max diameter 4 cm. For further clarification of the US findings, Computed tomography (CT) (Figure 1) and Magnetic Resonance Imaging (MRI) (Figures 2a, 2b & Figure 3) of the abdomen were also performed and revealed a homogenously hyper-intense multi-locular cystic lesion with max diameter 4 cm, located in the neck of the pancreas. Since it was difficult to conclude preoperatively whether the mass of interest corresponded to either a serous cystadenoma or a mucinous neoplasm or a cystadenocarcinoma we scheduled to perform a typical laparoscopic cholecystectomy in combination with laparoscopic evaluation of the mass in order to obtain a diagnosis and treatment if possible.

**Figure 4.**
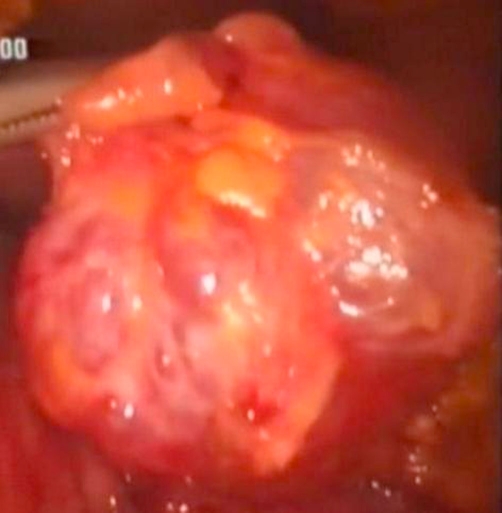
Laparoscopic macroscopic view of the serous cystadenoma at the pancreatic neck.

**Figure 5.**
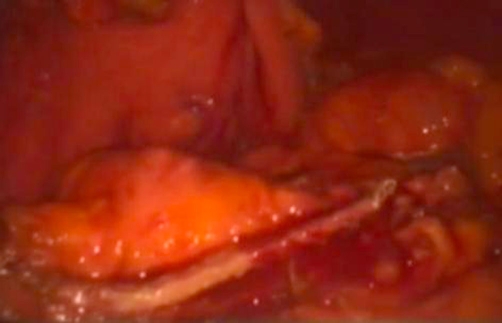
Laparoscopic view of the excision line at the pancreas after the resection of the pancreatic serous cystadenoma with ENDOGIA™.

Under general anesthesia with the patient in the supine position, a pneumoperitoneum was created by carbon dioxide insufflation and four trocars were placed in the typical locations for cholecystectomy. After typical laparoscopic cholecystectomy, a fifth 12 mm trocar was placed at the outer verge of *rectus abdominis* muscle, in order to ease further surgical manipulations. The gastrocolic ligament was divided by Ligasure™ vessel sealing system and a tumor of exophytic nature protruding from the pancreatic parenchyma was noticed at the upper pancreatic margin at the pancreatic neck. Because the tumor was quite mobile and lacked adhesions with the neighboring tissues, it was considered it could be excised en bloc laparoscopically without harming the main pancreatic duct. After dissection of the tumor, ENDO GIA™ was used for tumor excision from the pancreatic parenchyma and ENDO CATCH™ was used to collect and remove the mass from the peritoneal cavity (Figures 4 & 5).

Since the rapid histologic evaluation was negative for malignancy, a drain was placed in the excision area. The postoperative course was uneventful with a minor pancreatic leakage for the first two days. The drain was removed on the fourth day and feeding was started at the same day. The patient was discharged from the hospital seven days after surgery.

The standard histopathological study of the removed mass showed a serous microcystadenoma of the pancreas with no evidence of malignancy. There were not any late postoperative complications, while the patient remained asymptomatic and exhibited no sign of recurrence 6 months after the operation.

## Discussion

The cystic neoplasms of the pancreas are rare and represent a diverse spectrum of disorders, which have differences in the cells of origin, their biologic aggressiveness regarding the natural history and the malignant potential, and their management. In general they include four categories: (a). Serous cystadenomas, (b). Mucinous cystic neoplasms (MCNs; cystadenomas and cystadenocarcinomas), (c). Intraductal papillary mucinous tumor (IPMT, or mucinous ductal ectasia), and (d) Unusual cystic neoplasms, including the extremely rare cystic islet cell tumors (functional and nonfunctional), acinar cell cystadenocarcinomas, cystic choriocarcinomas, cystic teratomas, and cystic lymphangiomatous neoplasms [10].

Pancreatic serous cystadenomas are rare neoplasms, predominately affecting women (65%) at the seventh decade of life. Small neoplasms are usually asymptomatic, and the symptoms, most of which are non-specific, are caused primarily by a large space-occupying tumor mass. Systemic symptoms such as weight loss, malaise, anorexia, and fatigue are rare. The majority of the serous cystic neoplasms are found incidentally during the evaluation for unrelated intra-abdominal disorders or symptoms. They can be located anywhere in the pancreas [10].

A recent single-center study presented their experience of a large series of 106 patients with pancreatic serous cystadenoma [6]. In this study, it was observed a preponderance in female gender (75%) in the seventh decade of life, while almost half of the tumors were found incidentally. There was only a slight difference between proximal or distal location of the tumors; 44% were found in the head, neck or uncinate process of the pancreas versus 56% found in the body or the tail of the pancreas. Almost 50% of the patients were symptomatic presenting mainly with abdominal pain or fullness. Symptoms correlated with tumor size since tumors <4 cm were less likely to be symptomatic, but not with tumor location [6].

Most serous cystadenomas are microcystic composed of multiple small (<2 cm) cystic areas, forming a characteristic honeycomb-like appearance, which is observed grossly and on imaging, but also macrocystic variants have been rarely reported. In general, the improvement in abdominal imaging techniques (CT scanning, transcutaneous or endoscopic ultrasonography, and MR imaging) has offered the ability of non-invasive diagnosis with high accuracy in 90% of cases [6].

Since the majority of these tumors are benign, with only a few case reports of serous cystadenocarcinomas, the vast majority of the literature and most of the experts, suggest that the surgical resection of these tumors is recommended when they are large (more than 4 cm in maximal diameter) and symptomatic, or when the imaging techniques are unable to distinguish a serous cystic neoplasm from a mucinous lesion, which has greater malignant potential [6]. On the opposite, asymptomatic tumors with maximal diameter less than 4 cm should be clinically followed up together with serial imaging, perhaps every 2 years, for growth rate estimation [6].

When the cystic tumors are located in the body or tail of the pancreas a classical or laparoscopic distal pancreatectomy with or without splenic preservation can be performed [11,12]. When the tumors are located at the pancreatic head or neck traditionally pancreatico-duodenenectomy with classical Whipple’s procedure is required. However, limited resections, such as enucleation, duodenum-preserving pancreatic head resection, or median pancreatectomy, are possible when resecting small pancreatic tumors, to preserve as many functional parenchyma and structures as possible [12,13].

In our case, since pre-operative imaging studies including percutaneous US, CT, MRI could not accurately define the benign or malignant nature of the mass (serous or mucinus neoplasm of the pancreas), we decided to perform a typical laparoscopic cholecystectomy for symptomatic cholelithiasis combined with laparoscopic exploration and evaluation of the pancreatic tumor nature. With the laparoscopic procedure we had the opportunity to approach the mass, confine its resectability, and resect the cystic lesion at the same time.

There are some important issues regarding the optimal management of the serous pancreatic cystadenomas and our approach for the treatment of this neoplasm. Although the advances in abdominal imaging techniques have resulted into an increasing frequency of pancreatic cystic neoplasms identification they can not always accurately predict the benign or malignant nature of the cystic tumors of the pancreas [6]. Moreover, even when the diagnosis of serous cystadenoma is firm, there have been no data to date that allow one to safely predict the course of this tumor which may sometimes grow sufficiently to cause symptoms or have a malignant transformation during the lifespan of a given patient [14]. Consequently, despite the fact that we knew that the asymptomatic tumor was not very large (4 cm in diameter) we decided to resect the tumor, contrary to most experts’ recommendations and confirmed the benign nature of the mass (serous cystadenoma) before the completion of the operation.

There are improvements in pancreatic surgery (open or laparoscopic) with reduction of mortality rates, but the morbidity remains high and the consequences of loss of pancreatic tissue are serious [6]. In our case, the location and the protruding nature of the tumor, gave us the opportunity to resect the cystic mass from the pancreatic neck performing a laparoscopic method with minimal segmental resection of pancreatic parenchyma without harming the splenic vessels and the main pancreatic duct, while preserving the integrity of the whole pancreas. Otherwise we should perform a major resection performing open distal pancreatectomy with or without spleen-preserving or a more limited resection performing open or even laparoscopic central (median) pancreatectomy [13,15].

With this minimally invasive procedure we treated simultaneously the cholelithiasis and the cystic mass of the pancreas, avoiding two separate operations and perhaps a more invasive operation for this benign pancreatic lesion. With this technique we preserved the integrity of the whole pancreatic tissue since only a peripheral slide of pancreatic parenchyma was excised together with the mass, while maintaining the upper digestive and biliary anatomy and preserving the spleen. Furthermore there were no short- or long-term postoperative complications.

## Conclusion

Pancreatic serous cystadenoma is a benign tumor but imaging modalities are often difficult to distinguish it from malignant pancreatic cystic tumors. If the diagnosis of a serous cystadenoma is certain, surgery is recommended for large tumors and symptomatic patients, but if the diagnosis is questionable, it is better to resect the tumors under acceptable surgical risks, with the most limited and minimal method. We presented a case of pancreatic serous cystadenoma, which was treated with a novel minimally invasive laparoscopic method combined with standard laparoscopic cholecystectomy. In our opinion the laparoscopic resection is a feasible, safe and effective treatment of benign pancreatic tumors, in experienced hands under proper indications and optimal circumstances.

## Abbreviations

CT: Computed tomography
IPMT: Intraductal papillary mucinous tumor
MRI: Magnetic Resonance Imaging
MCNs: Mucinous cystic neoplasms
US: Ultrasound
